# Micromanagement of Immune System: Role of miRNAs in Helminthic Infections

**DOI:** 10.3389/fmicb.2017.00586

**Published:** 2017-04-13

**Authors:** Naina Arora, Shweta Tripathi, Aloukick K. Singh, Prosenjit Mondal, Amit Mishra, Amit Prasad

**Affiliations:** ^1^School of Basic Sciences, Indian Institute of Technology MandiMandi, India; ^2^Department of Immunology, Weizmann Institute of ScienceRehovot, Israel; ^3^Cellular and Molecular Neurobiology Unit, Indian Institute of Technology JodhpurJodhpur, India

**Keywords:** miRNA, parasite, helminth, immune-modulation, infection

## Abstract

Helminthic infections fall under neglected tropical diseases, although they inflict severe morbidity to human and causes major economic burden on health care system in many developing countries. There is increased effort to understand their immunopathology in recent days due to their immuno-modulatory capabilities. Immune response is primarily controlled at the transcriptional level, however, microRNA-mediated RNA interference is emerging as important regulatory machinery that works at the translation level. In the past decade, microRNA (miRNA/miR) research has advanced with significant momentum. The result is ever increasing list of curated sequences from a broad panel of organisms including helminths. Several miRNAs had been discovered from trematodes, nematodes and cestodes like let-7, miR155, miR-199, miR-134, miR-223, miR-146, and fhe-mir-125a etc., with potential role in immune modulation. These miRs had been associated with TGF-β, MAPK, Toll-like receptor, PI3K/AKT signaling pathways and insulin growth factor regulation. Thus, controlling the immune cells development, survival, proliferation and death. Apart from micromanagement of immune system, they also express certain unique miRNA also like *cis-*miR-001, *cis-*miR-2, *cis-*miR-6, *cis-*miR-10, *cis-*miR-18, *cis-*miR-19, trs-mir-0001, fhe-miR-01, fhe-miR-07, fhe-miR-08, egr-miR-4988, egr-miR-4989 etc. The specific role played by most of these species specific unique miRs are yet to be discovered. However, these newly discovered miRNAs might serve as novel targets for therapeutic intervention or biomarkers for parasitic infections.

## Introduction

The healthy state of an individual is governed by controlled regulation of various systemic processes and its protection from pathogens/allergens it encounters. This protection against pathogens is taken care by our immune system. The two arms of immune defense, innate and adaptive, work synergistically to protect host against pathogenic agents and to maintain immune homeostasis. One common strategy by which pathogens could challenge host immunity to promote their adaptive fitness is through the manipulation of crosstalk interactions between innate immune receptors/molecules. Extra-cellular parasites are smartest of the pathogens, which can evade immune system detection for a very prolonged period and can make conducive environment for themselves. Helminthic parasites, from the phylum Platyhelminthes (including trematodes and cestodes) and the phylum Nemathelminthes (nematodes) are the most common parasitic infectious agents affecting human being in tropical and developing countries. According to Global Disease Burden estimate approximately more than one billion people living in poor regions of sub-Saharan Africa, Asia and the Americas are getting exposure to one or more helminthic species (World Health Organization [WHO], 2012; Global Burden of Disease Study 2013 Collaborators, 2015). Apart from that, as a consequence of globalization and world trade, diseases are spreading from endemic to non-endemic regions of the world due to movement of human carriers. Unfortunately these parasitic infections fall in category of neglected diseases, despite producing a major economic burden on many developing countries health care system (Hotez et al., 2008; World Health Organization [WHO], 2012). Hence, off late massive effort is going-on to understand the immunology of these parasites. Apart from initial neglect of scientific community, lack of techniques to transfect parasites and make knockout strains to determine any effects of specific gene on the host response or on parasite survival make host-parasite interactions study more challenging. Due to this void of knowledge to the helminths infections there is paucity of information on host–parasite interactions, factors regulating inflammatory pathology and protective immunity against these parasites.

Helminths infection and the resultant host immune response is consequence of an extended vibrant co-evolution between the host and parasite with constant fine-tuning. For parasites it is a necessity to trick the host from developing an effective immune response, to find a right niche for maturation and propagation and to do so without killing or excessively harming the host. Conversely, the host has to generate an effective immune response to expel the parasite and minimize its harmful consequences, at the same time not damaging its own tissues and compromising its ability to effectively respond to other pathogens. The host mechanisms of defense against these multicellular parasites range from primary barriers to the most elaborate signaling mechanisms, which involves different kind of cells and molecules, which are capable of non-specific and specific recognition and elimination of invasive agents. All immune evasion mechanisms are extremely embedded in the fine details of the molecular machinery that regulate the immune response. These parasites have evasion mechanisms in place that depends upon several factors like life cycle stage, route of infection and the microenvironment in which they survive in host etc. How higher vertebrate immune system fights off/or learns to live with worm infections has been a hot topic for long and extensively studied, but many questions remain unanswered.

Recently, whole genome project for several bacteria/fungus/parasites of clinical and veterinary significance have been completed, which had provided a new area of study, i.e., role of small non-coding RNAs (siRNA, miRNA/miR, and piRNA). These small RNAs range from 18 to 30 nucleotide in length and are crucial regulators in the silencing of target genes in a wide range of metazoans, plants, and fungi (Bartel, 2004, 2009; Molnar et al., 2010). Among them, miRs are a class of small non-coding RNAs that negatively regulate gene expression by directly binding to the 3′ untranslated region (3′ UTR) of their mRNA targets (Bartel, 2004; Krol et al., 2010; Zhao et al., 2014). They were first discovered in a nematode worm *C. elegans* in 1993, where the gene expression of lin-14 was found to be down regulated by miRNA lin-4 (Lee et al., 1993; Wightman et al., 1993). Thus finding was initially taken as an oddity in worms. However, discovery of miRNA in mammals established it as fundamental mechanism of regulation of gene expression. A number of miRs have been discovered in plants and animals. They are evolutionary conserved sequences, they play crucial role in embryogenesis, development and regulation of various systems. miRNAs silence the transcript by complementary binding to mRNA, repressing translation or by decreasing target mRNA levels. They are considered as key mediators in immune system and are involved in development, activation and regulation of immune cells. By controlling fine-tune of protein expression, miRs can contribute to regulatory circuits by providing quantitative control of gene output, i.e., protein. Particularly, they often exercise their influence by regulating dosage-sensitive genes for which small fluctuations in protein expression may contribute to a significant change in functional output. Their role has been established in a number of infectious, autoimmune and metabolic diseases (Rottiers and Näär, 2012; Garo and Murugaiyan, 2016). However, very little is known about the role of miRNA in helminthic infections and especially in the development and function of immune cells. List of miRNAs involved in helminthic infections of human importance is given in **Table 1**. In this review, we had discussed about the importance of miRNAs in helminthic infection and how these miRNAs either host derived or parasite derived may influence the outcome of infection.

**Table 1:** List of miRNAs involved in helminthic infections of human importance identified from parasite and host.

Parasite	Parasite derived miRNA having immuno-regulatory function			Human miRNA
	In parasite	In Host	Novel miRNA	
*Schistosoma japonicum*	sja-miR-125b sja-miR-190 sja-bantam sja-miR-36 sja-miR-124 sja-miR-36 sja-miR-7 sja-let7	sja-miR-277 sja-miR-3479	sja-let-7 sja-miR-71 sja-bantam	miR-223 miR-155 miR-146a/b miR-122 miR-21 miR-34a
*Fasciola hepatica*	fhe-mir-71a fhemir190 fhe-mir-1 fhe-mir-125a		fhe-miR-01 fhe-miR-07 fhe-miR-08 fhe-miR-10	
*Clonorchis sinensis*	*cis-*miR-16 *cis-*miR-93 *cis-*miR-95 *cis-*miR-136 *cis-*miR-153 *cis-*miR-195 *cis-*miR-199a		*cis-*miR-001 *cis-*miR-002 *cis-*miR-006 *cis-*miR-010 *cis-*miR-018 *cis-*miR-019	
*Echinococcus granulosus*	egr-miR-2 egr-miR-71 egr-let-7 egr-miR-10 egr-miR9 egr-miR-277 egr-miR-125		egr-miR-4988 egr-miR-4989 egr-miR-4990 egr-miR-4991	
*Mesocestoides corti*	mco-miR-Bantam mco-miR-let-7 mco-miR-10 mco-miR-71 mco-miR-4989		mco-miR-12067 mco-miR-12068 mco-miR-12068 mco-miR-12069 mco-miR-12070 mco-miR-12071	
*Ascaris suum*	asu-miR-391 asu-miR-404		asu-miR-391 asu-miR-404	
*Angiostrongylus cantonensis*	aca-miR-71 aca-miR-1 aca-miR-60 aca-miR-1 aca-miR-77 aca-miR-44	aca-miR-124	IV_2 IV_4 IV_25	miR-146a miR-132 miR-155 miR-206 miR-223 miR-511
*Trichinella spiralis*	trs-miR-1 trs-miR-let-7 trs-miR-72 trs-miR-87 trs-miR-124		trs-miR-0001	

Dicer is a RNase III enzyme that cleaves precursor miRNA (pre-miRNA) into miRNA duplex and Argonaute is the major executor of miRNA/siRNA function, respectively. Several protozoan parasites (e.g., Plasmodium spps) lack coding genes for the key miRNA processing enzymes DICER and Argonaute, hence are not capable of synthesizing their own miRNA (Rathjen et al., 2006). But these parasites exploit miRNA pathways of host cells by actively communicating with the host cells via exosomes and microvesicles (Militello et al., 2008). The evasion mechanisms at post-transcriptional level by the means of miRs of host and parasite and their interaction will help in understanding the survival strategy of the parasites at molecular level.

## miRNA in Immune Activation: Strategic Decision Makers

**FIGURE 1:**
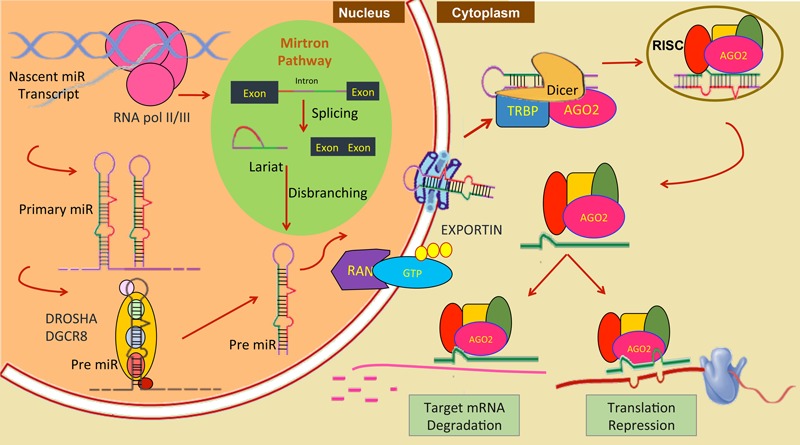
**Nuclear event in miRNA biogenesis and its function in canonical pathway and mitron pathway (green circle) in animals**.

At present, by using *in-silico* tools like mireap, miRDeep2, miRAnalyzer or by homology mapping, miRNAs have been computationally or experimentally investigated in more than 35 helminthic parasites (11 trematodes, 8 cestodes, and 16 nematodes, including two plant parasitic nematodes, *Bursaphelenchus xylophilus* and *Globodera pallida*) more than 620 pre-miRNAs are currently listed at miRBase^1^ of parasitic origin. The mature miR transcripts are result of elaborate processing (Bartel, 2004; Krol et al., 2010) and can be divided into canonical and non-canonical pathways. Most ∼22 nt mammalian miRNAs come from canonical pathway. It’s synthesis begins in the nucleus, where the RNA Pol II transcribes the miR gene resulting in long polyadenylated mRNA like sequence. In the first processing step, Drosha and RNase III enzymes act on it producing pri-miR, which is a 70 nt long hairpin structure. miRs are then transported out to cytoplasm by Exportin-5 and RAN-GTP proteins. In cytoplasm, another RNase III enzyme “DICER” acts on the pri-miR transcript resulting in miR-duplex with one guide strand and another miR^∗^ strand. The guide strand is loaded into the RISC (RNA induced silencing complex) which targets mRNA for degradation or translation repression and miR^∗^ is degraded. Which strand will be retained depends on the relative thermodynamic stability of the two ends of the duplex intermediate (Khvorova et al., 2003). The biogenesis of miRNA and its different steps are shown in **Figure 1**. For miRNA coming from non-canonical pathway, they are not dependent on Drosha for cleavage, instead they undergo splicing and are further stabilized by Ago2 protein bypassing Dicer (Cheloufi et al., 2010). It’s a complex network where many proteins are involved and not all molecules have been identified. A single mRNA can be regulated by more than one miR and one miR can regulate more than one mRNA. Some miRs are packed in exosome vesicles and transported to nearby cells, this confer protection to miR against cellular degradation.

The first indication that miRNA might regulate the immune responses came from a report in 2004, showing selective expression of miR-142a, miR-181a, and miR-223 in immune cells (Chen et al., 2004). Since then many miRNAs have been implicated in the regulation of maturation, proliferation, differentiation and activation of immune cells. Recent studies indicate that these miRNAs (miR-17–92 cluster, miR-150, miR-155, miR-181, miR-206, miR-223, and miR-511 etc.) are selectively and/or highly expressed in immune cells like macrophages, B-cells, T-cells, monocytes etc., and they are directly involved in cell development and immune regulation (Johnnidis et al., 2008). Even the role of TLRs in activation of miRs had been identified in epithelial cells and in whole animal studies. These studies had shown that the activation of TIRs and TNF-α receptor results in rapid expression of a host of miRNAs including let-7, miR-9, miR-99b, let-7e, miR-125a, miR-132, miR- 146a, miR-146b, miR-155, miR-187, and miR-223 (Taganov et al., 2006; Tili et al., 2007; Bazzoni et al., 2009; Ceppi et al., 2009). Emerging data have identified important role of miRNAs to the development and function of innate immune cells. Granulocytes formation (granulopoiesis) is found to be blocked in miR-21 and miR-196 depleted bone marrow as these miR interacts with transcription factor growth factor independent 1 (GFI1) (Velu et al., 2009). Similar to granulopoiesis, several studies had shown that transcription factors involved in monocytopoiesis are regulated by, and/or specific miRNAs, which indicates a connection between these molecular species during development (Fontana et al., 2007; Rosa et al., 2007). miR-155 and miR-223 are differentially expressed in hematopoietic cell lineages and regulate myeloid progenitor cells (Gilicze et al., 2014). Similarly now, several miRs had been associated with various infections, and macrophage inflammatory response to infection involves the upregulation of numerous miRNAs, such as miR-155, miR-146, miR-147, miR-21, and miR-9. miR-155 upregulation indicate an active immune response involving activation of macrophages and proliferation and differentiation of T cell and B cell (Tsitsiou and Lindsay, 2009) post-infection conferring protection against the parasite. Whereas miR-233, which negatively regulates Roquin ubiquitin ligase, eventually regulates IL-17 mediated inflammation, IL-17 is upregulated in bacterial infection, severe sepsis and septic shock (Wang et al., 2012). miR-124 had been found to be involved in regulating macrophages activation and suppressing microglia in CNS (Ponomarev et al., 2011). A detailed list of miRNAs identified in immune cells and their target gene are given in **Table 2**.

**Table 2:** List of miRNAs with their roles in immune system.

miRNA	Cell type where it is expressed	Target gene
let-7e	Macrophages	TLR4
let-7c	HSCs, Macrophages, iNKT cells	APC2, WNT1, PLZF
miR-9	Myeloid cells	NFKB1
miR-10	HSCs	HOX family
miR-17-5p-20a-106a	Myeloid cells	RUNX1
miR-21	Myeloid cells	PTEN, PDCD4, IL12A
miR-34	Dendritic cells and B cells	FOXP1, WNT1, and JAG1
miR-125b	HSCs	SMAD2, SMAD4, TGF-β
miR-126	HSCs	HOXA9 and PLK2
miR-142-3p	Treg cells	AC9
miR-146	Monocytes	IRAK1, IRAK2, and TRAF6
miR-150	B cells, T cells	MYB
miR-155	B cells, T cells, Macrophages, DCs	SHIP1, SOCS1, C/EBPβ, BACH1, CSFR, TAB2
miR-181a	T cells, NK cells	PTPN22, DUSP6, DUSP5, NLK/Notch
miR-196b	HSCs	HOX family
miR-221/miR-222	HSCs	KIT
miR-223	Myeloid cells, Macrophages	MEF2C, IGF1R
miR-326	T cells	ETS1
miR-424	Myeloid cells	NFIA, SPI1
miR-451	Macrophages	RAC1

*AC9, adenylate cyclase 9; *BACH1*, BTB-, CNC- and b ZIP-domain-containing transcription factor; *BIM*, BCL-2-interacting mediator of cell death; *CEBPB*, CCAAT/enhancer binding protein-β; *CSFR*, colony-stimulating factor receptor; DC, dendritic cell; *DUSP*, dual-specificity protein phosphatase; *FOXP1*, forkhead box P1; HOX, homeobox; HSC, haematopoietic stem cell; IGF1R, insulin-like growth factor 1 receptor; *IL12A*, interleukin-12A; *IRAK*, IL-1R-associated kinase; *JAG1*, Jagged1; *MEF2C*, myeloid ELF1-like factor 2C; miR, microRNA; *NFIA*; nuclear factor I/A; *NFKB1*, nuclear factor-κB subunit 1; *NLK*, nemo-like kinase; *PDCD4*, programmed cell death 4; *PLZF*, promyelocytic leukemia zinc finger protein; *PTEN*, phosphatase and tensin homolog; *PTPN22*, protein tyrosine phosphatase, non-receptor type 22; *RAC1*, RAS-related C3 botulinum substrate 1; *RUNX1*, runt-related transcription factor 1; *SHIP1*, SH2-domain-containing inositol-5-phosphatase 1; *SHP2*, SH2-domain-containing protein tyrosine phosphatase 2; *SOCS1*, suppressor of cytokine signaling 1; *TAB2*, TAK1-binding protein 2; *TLR4*, Toll-like receptor 4; *TRAF6*, TNFR-associated factor 6; T_*Reg*_ cell, regulatory T cell.*

The miR-146 had been identified as regulator of inflammation in CNS by interacting with IRAK at 3′UTR and plays role in NF-κβ signaling with the help of adaptor TRAF6 (Yu et al., 2014a) and modulates TNF-α expression in mice model of meningitis (Mo et al., 2016). Similarly, higher expression of miR-155, miR-206, miR-223, and miR-511 had been co-related with higher inflammation in mice model of meningitis (Yu et al., 2014b). Role of miR has also been identified in altering the tumor progression and metastasis. High throughput miR profiling had identified upregulated miR-511 in alternatively active macrophages in solid tumors, suggesting their essential role in M2 macrophages differentiation as well as in the pathology of the disease (Squadrito et al., 2013). Thus it is clear that miRs are crucial for immune activation and appropriate immune response.

## miR in Trematode

Among the neglected tropical disease, a huge amount of disease burden lies on the disease caused by the flukes, especially digenetic trematodes such as *Schistosoma* spp., *Fasciola* spp., *Clonorchis sinensis, Paragonimus* spp., *Eurytrema pancreaticum, Fasciolopsis buski, Philophthalmus lacrimosus* etc. Depending on the infection site they can be tissue flukes or blood flukes. They need a mollusc and a vertebrate to complete their life cycle. Not much information is available about the miRNA present in these species except schistosoma, which is a model organism for this family.

### Schistosoma

Blood flukes *Schistosoma japonicum, S. haematobium*, or *S. mansoni* cause schistosomiasis also known as bilharzia, it affects nearly 230 million people worldwide (Vos et al., 2013; Colley et al., 2014). Snails are the intermediate host for this parasite and mammals are definite host, in which they parasitize blood capillaries of mesenteries or bladder plexus (depending on species) after penetrating the skin. The immune evasion strategies adopted by this parasite have been previously reviewed (Keating et al., 2006). As per the existing miR database (vesrion 21), 79 mature miRNAs in *S. japonicum* and 225 mature miRNAs in *S. mansoni* have been reported. The miRNA profile varies for male and female parasite, thus suggesting their role in morphogenesis, development and reproduction (Marco et al., 2013). Significant differences in expression profile of cercarial and schistosomula lung stage were also observed, indicating role of stage specific miRNA in development (Cai et al., 2013a). A comparision of pre- and post-Schistosoma infection in mice model had identified change in expression of more than 130 miRNAs (Cai et al., 2013b). Cai et al. (2013b) also observed a significant upregulation of the mmu-miR-146b, mmu-miR-155, mmu-miR-223, mmu-miR-142-3p, mmu-miR-15b, and mmu-miR-126-5p after 30 dpi. Out of these miRNA, miR155, miR223 and miR146 are already known as suppressor of Toll-like receptor and cytokine signaling via a negative feedback regulation loop involving down-regulation of IL-1 receptor-associated kinase 1 (IRAK1) and TNF receptor-associated factor 6 (TRAF6) protein levels (Taganov et al., 2006). Thus suggesting their role in restricting hepatic pathology at mid and late stage of infection. miR-155 is also associated with transcription factor c-Maf and it attenuates Th2 response in CD4+ T-cells (Rodriguez et al., 2007). In a mice model study of infected liver cells, highly elevated expression of miR-34c, miR-199, miR-134, miR-223, and miR-214 at 45dpi had been noticed (Cai et al., 2013b). Similarly in another murine model study, Han et al. (2013) had identified 220 miRNAs in different tissues of the BALB/c mice after 45 days of infection with schistosoma. They identified 44 upregulated miRNA; eight miRNAs in liver, eight in spleen and 28 in the lungs, and 36 down regulated miRNAs; three miRNAs in liver, five in spleen and 28 in the lungs. They also did functional analysis of these miRNA and found these are primarily associated with immune regulation, nutrient metabolism, cell proliferation and differentiation and molecular regulation. The miR-214 and miR-199 showed maximum differential expression. They are found upregulated in liver fibrosis and mediate TGF-β signaling. TGF-β plays important role in embryogenesis and development of Schistosoma (Freitas et al., 2007; Okada et al., 2015).

Study of parasite-derived miRNA had opened several new avenues apart from understanding the pathogenesis of disease, providing new drug targets, these cellular miRNAs have potential to be a reliable biomarkers of infection as their levels were found to be elevated in serum as well in tissues, but only after 12 weeks post-infection. Hence, they may not be suitable for progressive pathology. But the expression of parasite derived miRs like miR-277, Bantam, miR-3479-3p were found significantly high in serum in *S. mansoni* infection and miR-10-5p, miR-3479-3p and Batnam in case of *S. japonicum*, and can be used as biomarkers with improved sensitivity for differentiating egg positive and negative individuals (Cheng et al., 2013; Hoy et al., 2014). de Souza Gomes et al. (2011) applied whole genome computational approach and identified 67 mature and 42 precursor novel miRNA in schistosoma, among them 26 precursors and 35 mature miRNAs were found to be evolutionarily conserved. Out of these small miRs, several have potential to interact with smad2 to regulate TGF-B signaling, thus have the potential to influence Th1 immune activation, however, this need to be verified by other means. miRNA has been tested for diagnostic purpose in schistosomiasis with great accuracy (Hoy et al., 2014) as the currently used microscopic stool examination or serum based assay have poor sensitivity in detecting low-intensity infections and fail to detect where eggs are trapped in tissues and not excreted or patient had infection with other helminth (Stothard et al., 2011). Hoy et al. (2014) found miR-277, miR-3479-3p, and Bantam from schistosoma could detect infected individuals from low and high infection intensity sites with specificity/sensitivity values of 89%/80% and 80%/90%, respectively.

### Clonorchiasis

This food-borne carcinogenic liver fluke causes infection in human called Clonorchiasis. Approximately 35 million people are globally infected by this parasite, especially in Asian countries like China, Japan, Vietnam and Korea (Lun et al., 2005). The liver fluke has two intermediate hosts: mollusks (first, infected by parasite eggs) and fish (second, infected by larvae). Humans get infected with this parasite when they consume these infected fish raw or without proper cooking. It has been classified as group1 biological human carcinogen by the International Agency for Research on Cancer (Shin et al., 2010). To identify the miRNAs present in adult *C. sinensis*, deep sequencing and bioinformatics extrapolations combined with stem-loop real-time PCR analysis approach was applied (Xu et al., 2010), they identified a total of 62,512 conserved miRNAs which belonged to 284 families along with six novel miRNA (*cis-*miR-001, *cis-*miR-2, *cis-*miR-6, *cis-*miR-10, *cis-*miR-18, and *cis-*miR-19). Among the conserved family of miRs identified by this approach, miR-71 was having the highest number of reads followed by mir-277 and miR-215, suggesting their role in parasite’s survival and metabolism inside the host. These parasite derived novel miRs can be used to assess the cancer risk associated with liver fluke infection. In an *in-vitro* study using H-69 lung cancer cell line, Pak et al. (2014) reported upregulation of miR-16-2, miR-93, miR-95, mir-153, mir-195, miR-199a-3p, and down-regulation of miR let7a, let7i, miR-124a in the presence of excretory secretory protein of *C. sinensis*. Among the miRNAs, seven of them (miR-16-2, miR-93, miR-95, miR-136, miR-153, miR-195, and miR-199a-3p) were mainly associated with esophageal adenocarcinoma, breast cancer and colorectal carcinoma, suggesting the role of these miRs in cell-proliferation and cell-signaling.

Recently, Yan et al. (2016) compared the hepatic miRNAs expression profiles from *C. sinensis* infected mice at 2, 8, and 16 weeks post-infection using miRNA-microarray and validated it by quantitative real-time PCR (qRT-PCR). They noticed 349 miRNAs were differentially expressed during infection, out of which 143 were down-regulated and 206 were up-regulated. These all dysregulated miRNAs were potentially involved in the pathological processes of clonorchiasis by regulating various signaling pathway like cancer-related, TGF-β, MAPK, TLR, PI3K/AKT signaling pathways. Interestingly 169 of these dysregulated miRNAs were predicted to be involved in the TGF/Smads signaling pathway which plays an important role in the biliary fibrosis caused by *C. sinensis*.

### Fasciolosis

The common liver fluke, a flat worm is now considered as emerging zoonotic disease. The two species *Fasciola hepatica and F. gigantica* causes fasciolosis in mammals. It completes its life cycle in its intermediate-host snail and definitive-host mammals. It is estimated that worldwide 2.4–17 million people are infected with Fasciola spp. and another 180 million are at risk of infection in Americas, parts of Europe, South Africa, the Middle East and Asia (Keiser and Utzinger, 2005). This infection is of zoonotic importance and miR regulation play crucial role in pathogenesis of this disease. Xu et al. (2013a) used next generation sequencing, bioinformatic analysis and stem-loop qRT-PCR to identify the miRNA expressed by these two parasites. The comparative miR expression profile of these two species had revealed 13 species-specific miRs that matched with miR4006b of *Ciona intestinalis* and miR 1957 matched with *Mus musculus*. Other miRs that could map to metabolic regulation and catalytic and binding function were common to both species (Xu et al., 2013b). Fromm et al. (2015) used deep-sequencing method and identified 54 miRNA (42conserved and 13 novel) and isolated miR from extracellular vesicles (EV) also. They found fhe-mir-125b as most abundant miRNA in EV; this miR belongs to the eumetazoan mir-10 family and has three orthologs in the host (cattle; *Bos taurus*; bta-mir-125a, bta-mir-125-1/-2b). Human mir-125 family members have been previously identified as decisive players in immune system development, immunological host defense and in cancer (Sun et al., 2013). Apart from this, they also found striking similarities between fhe-mir-2b-A and fhe-mir-2a-B and the two mir-27 orthologs, bta-mir-27a and bta-mir-27b, these two are known to target the 3′ un-translated region (UTR) of myostatin and insulin growth factor (IGF) in cattle and human (Miretti et al., 2013). While myostatin down-regulation has been associated with increased liver cell proliferation (Watts et al., 2014) and the decreased of has been associated with increased cancer risk (Loumaye et al., 2015).

## miR in Nematode

The tube within tube organisms are also called as round worms and belong to class nematode. They have several human infecting members and are cause of a number of common parasitic infections like ascariasis, filariasis, trichinellosis, angiostrongyliasis, strongyloidiasis etc. As per the recent updates by WHO these soil worms affect 24% of population worldwide and thus bears zoonotic importance and are crucial parasites which need extra attention of scientific world. Incidentally, the first miRNA was discovered in a nematode *C. elegans*.

### Ascariasis

The roundworm nematode *A. suum* and *A. lumbricoides* (largest known worm) causes infection in pigs and humans. Approximately 807 million people are infected and 4.2 billion are at risk of infection globally (de Silva et al., 2003). Ascaris don’t involve any intermediate host, the fertile eggs are deposited on soil/plants and humans get infected when we ingest the infected soil/plant part. The eggs develop into larvae, which can penetrate the wall of duodenum to reach blood stream and parasitize at lungs, liver or heart. Only few papers are available about this parasite hence very little is known about the role of miR in this infection due to negligence and late availability of whole genome sequence of this parasite. Wang et al. (2011) did deep sequencing study which showed Ascaris spp. has similar pathway dependence as in *C. elegance*. Their germline is siRNA pathway dependent, whereas embryogenesis and larval development is dependent on miR pathway. The miR expression profile of male and female worm shared two novel miRs Asu-miR-share-391 and Asu-miR-share-404, the expression of miR-391 was more abundant in male than in female (Xu et al., 2013b). But their role in immune regulation needs to be further investigated.

### Trichinellosis

*Trichinella spiralis* is a food borne intracellular helminth that causes trichinellosis in animals. Humans get infection by ingestion of undercooked pork infected with its larvae (Murrell, 2013) and it encyst in the muscle. Its infection is repotrted from approximately 55 countries across the world including European countries (Pozio, 2007; Murrell and Pozio, 2011). The parasite remains asymptomatic and harbors in host tissue, the immune modulation is not only against the parasite but also against the auto antigens and allergens and also suppresses the malignant cells (Wang et al., 2009; Park et al., 2011). Chen et al. (2011a) used deep sequencing combined with stem-loop real-time polymerase chain reaction (PCR) analysis to identify the miR present at larval stage of *T. spiralis*. They analyzed the sequence against *C. elegans* and *Brugia malayi* miRdataset as reference and identified 11 and 12 conserved miRNA, respectively, with one unique miR. The miR expression profile showed miR-1, let-7, miR72, miR87 and miR-124 were highly abundant in the muscle larvale, suggesting it’s potential role in growth and metabolism. The one novel miR identified was trs-mir-0001 and it’s role in regulation and as signature miR for Trichinellosis needs to be further studied.

### Angiostrongyliasis

The parasitic nematode *Angiostrongylus cantonensis* also known as Rat Lungworm is a prime causative agent of eosinophilic meningitis and eosinophilic meningoencephalitis in humans (Alicata, 1965). Inside the human host it penetrates the intestinal wall, infects the CNS by entering the blood stream and breaking the blood brain barrier that causes a lot of neuronal damage. As is the situation with other helminthic parasite, very few researches has been undertaken in this direction so far and no novel miR has been reported. Chen et al. (2011b) studied the miR profile among adult male and female *A. cantonensis*. They found miR-71, miR-1, and miR-60 were abundant in male and female parasites but the relative expression or copy number was higher in males, implying their role in adult maturation and development. But they could not identify any novel miR. There were stage specific miR expression observed in young and adult worm, though the miR-1, miR-77, and miR-44 were abundant in young and adult worm stage suggesting they might be crucial for the survival (Chang et al., 2013). As the infection causes damage in CNS the inflammation in astrocytes is related with higher expression of miR-146a and miR-132, these miRs have been previously reported to be associated with chronic inflammation in neurological disorders, innate immunity regulation and inflammation regulation (Shaked et al., 2009; Iyer et al., 2012; Xie et al., 2013).

## miR Host Immune Modulation in Cestode

Cestodes are parasitic flatworm causing chronic zoonotic diseases. They include organisms like *Echinococcus* spp., *Taenia* spp., and *Diphyllobothrium* which generally cause asymptomatic intestinal infection which can be sometimes associated with mild abdominal pain or nausea. Their larval stage can penetrate through intestinal mucosa and travel to elsewhere causing infection with varied pathology.

### Echinococcosis

The causative agents of echinococcosis or hydatid are *Echinococcus granulosus, E. multilocularis*, and *E. canadensis*. These cestode parasites complete their life cycle in two mammalian hosts, ungulates as intermediate host and humans happen to be accidental host. Two of them, *E. granulosus* and *E. multilocularis* are known to cause most of the human infections. The miR expression profile of *E. granulosus* was studied for its different developmental stages and the protoscolex and cyst wall both showed the expression of miR-2, miR-9, miR-10, miR-27, let-7, and miR-71 except for miR-125 which was present only on protoscolex (Cucher et al., 2011). These miRs are involved in metamorphosis of parasite along with let-2. The protoscolex of *E. granulosus* had higher expression of miR-2 and miR-125 and their sequence showed variability, suggesting that it is due to binding to various 3′UTR targets. Interestingly, four novel miRs have been also identified in this parasite, namely miR-4988, miR-4989, miR-4990, and miR-4991. In a recent miR profiling in *E. canadensis* and it’s comparison with *E. granulosus* for identification of conserved miR and novel miR it was shown that let-7 and miR-71 were found to be highly abundant and accounted for about 50% of whole miR expression levels indicating role of these miRs in parasite’s survival in the intermediate host. miR-9 was also found to be highly expressive and miR-4989 expression appeared only in cyst wall samples. The miRnomes of echinococcus species showed expression of conserved miR profiles mir-36, mir-67, mir-92, mir-184, mir-281, miR-307, mir-1992, and miR-3479 which were previously thought to be lost in Echinococcus (Fromm et al., 2013; Jin et al., 2013; Cucher et al., 2015; Macchiaroli et al., 2015; Kamenetzky et al., 2016). The miR-10, let-7, miR-71, miR-4989, and Bantam hold the potential for diagnostic biomarkers for this infection as they are highly expressed in metacestodes of echinococcus species, though further experiments need to be undertaken to validate their role (Cucher et al., 2015; Macchiaroli et al., 2015). In a study involving miR-71 mimics it revealed that this Echinococcus multilocularis miR-71 (emu-miR-71) mimics significantly reduced the production of Nitric Oxide (NO) in *in vitro* experiment on RAW267.4 cells after 12 h of treatment (Zheng et al., 2016). The role of NO in echnicoccosis in host immune modulation has been previously described (Zheng, 2013). It does this by increasing the expression level of proteins Ago 1 and Ago 4 which are suggested to increase the stability of RISC complex. The exosome derived parasitic-miR-71 can be further used to understand exact mechanism underlying pathogen-host interaction (Buck et al., 2014; Zheng et al., 2016). The intestinal miR profiling of Kazakh sheep infected with *E. granulosus* eggs revealed an array of miRs for resistant and non-resistant haplotypes to infection. The resistant haplotypes had differentially expressed miR-21-3p, miR-134-5p, miR-542-5p, and miR-671 these miRs were found to be associated with NFκβ pathway and increased resistance to infection suggesting their role in inflammation, though further functional analysis needs to be undertaken (Jiang et al., 2016).

### Taeniasis

*Mesocestoide corti* is helminthic parasite that infects rats/mice. Though it does not infect human being it is used as a model organism for studying immunopathogenesis of class cestoda, of which Taenia is most important genus having mammals as host and *T. solium* is the most important parasite as it infects human. Larvae of taenia infect central nervous system causing neurocysticercosis, which is one of the most common cause of acquired epilepsy in developing world (Garcia et al., 2005; Prasad et al., 2008). *M. corti* have two life stages, tetrathyridia larvae and strobilated worm, and recently both were exploited for expression study of the miRNAs. The stage specific novel miR like miR-12071 was found in tetrathyridia stage and three conserved miRs found in storbilated worms were miR-2b, miR-7a, and miR-3479b. Besides these novel miRs there were certain common miRs and are highly expressed in both stages like bantam-3p, let-7-5p, miR-10-5p, miR-71-5p, and miR-4989-3p (Basika et al., 2016). The significance of miR-71 in *Echinococcus* spp. has already been discussed, its expression is found abundant in *M. corti* as well. The miR-71 in *C. elegance* has been shown to be associated with increased longevity via insulin-receptor/phosphatidylinositol 3-kinase pathway (Boulias and Horvitz, 2012). But whether the same mechanism occurs in *M. corti* needs further investigation. The up-regulated miR-36b also found in Echinococcus is associated with regenerative potential in planarians (Friedländer et al., 2009) and rapid proliferation in Echinococcus (Cucher et al., 2015) in *M. corti* tetrahyridia stage this miR is thought to control proliferation by asexual reproduction (Basika et al., 2016). The miR profile showed similarly expressed miRs in Echinococcus. The miR-125 and miR-2 in the storbilated stage suggests their role in correct sexual development via notch signaling (miR-125). It’s difficult to comment on the status of lost conserved miR families as some did match to echinococcus and the ones found in *M. corti* were absent in Echinococcus. *M. corti* expression profile reveals lots of potential miRs for further understanding of biological processes. Further assessment of post-transcriptional regulation of these miR in host-parasite interaction will enable a better understanding of class cestoda.

## Discussion

Helminthic infections are receiving great attention off late, due to their wide spread infection and newly established role as novel regulators of development, sex differentiation, drug resistance and immune modulators. Hence, microRNAs present in these parasites are also getting increasingly higher attention from research community to understand their role in immune modulation and auto-immune disorders and to explore their therapeutic potential.

**FIGURE 2:**
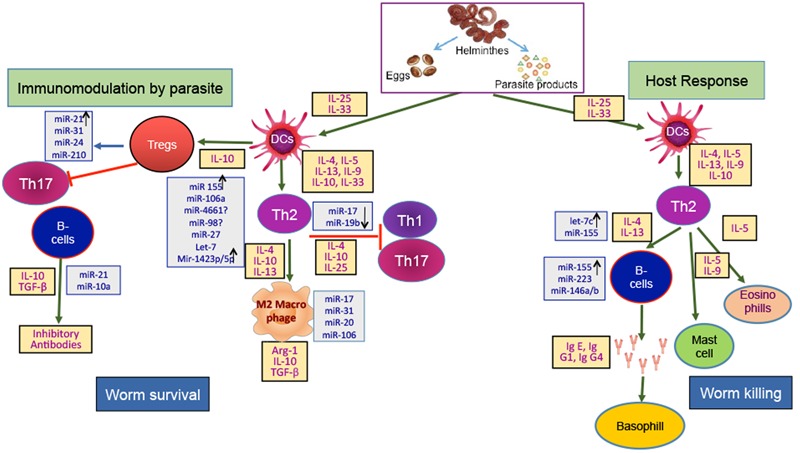
**Host-Helminth interaction: There are two sides of immune response against a parasite invasion, one leads to worm survival in host and the other to worm killing.** The breaking of intestinal epithelial barrier by helminth generate alarmins and recruit dendritic cells to the site of infection. Helminth’s associated immunogens like excretory secretory (ES) products, eggs, helminth derived products etc., plays important role. The immunomodulation by helminth and host response have specific cytokine signature. The immune regulation from worm survival to worm killing is tightly regulated at post-transcriptional level by microRNA. Immune cells and cytokines have specific microRNA profile which decide the course of pathway. Here, microRNAs stated in gray boxes have been found in host of diseases and are suspected to play role in host-helminth interaction by controlling the immune cells or associated cytokines.

By now, several studies had indentified different miRNA expression in various parasitic infections and the number of parasitic diseases in which the role of miRNAs has been confirmed is continuously increasing. The emerging role of miR in the helminthic diseases had brought a range of novel miRs, circulatory miRs and differentially expressed miRs at various life stages of helminths. miRNAs are small and comprise of a known sequence that is often completely conserved across species, which makes it a very attractive features from a drug development standpoint. A lot of conserved miRs are found across different classes, these miRs could give molecular cues of the processes common to all and hence can be used as broad range targets for entire class. The important and varied roles of miRNAs had identifid these molecules as valid targets for intervention strategies. However, targeting miR has its own challenges, as evident from the profiles of immune responsive miRs like miR-155, miR-146a/b etc., they are common host’s responsive miRs but it’s difficult to say if they have a common target or follow the common signaling mechanism as one miR can have multiple targets. microRNAs can serve as good and reliable biomarkers due to their high stability in paraffin-embedded tissues from clinical samples or in human plasma raised the possibility that miRNA expression analysis may be a useful tool to define disease states (Nelson et al., 2004; Mitchell et al., 2008). Currently, there are microRNA panels available, which are helping physicians in determining the origins of cancer in disseminated tumors (Nelson et al., 2004). In addition to cancer, miRNA expression profiles could also be used to distinguish distinct forms of heart disease (van Rooij et al., 2006), muscular disorders (Eisenberg et al., 2007) and neurodegenerative diseases (Hébert and De Strooper, 2009). However, at present there is no miRNA based clinical tool is in use for helminthic infections. We have summarized and suggested the role of miRNA in helminthis infection in **Figure 2**. As there are still several concerns to be addressed regarding the complete biological functions of miRNAs in the development/growth/maturation of parasitic helminths and its contribution in the formation of host pathology. Another major unanswered question is whether inhibitors of worm-specific miRNAs can kill parasitic helminths? And if yes, what will be commercial viability of that drug, as at present helminthic infections are largely treated by cheap protein inhibitor based drugs.

Development of microRNA-based therapeutics has proved more challenging than anticipated. The slow progress mainly stems from the general technical challenges with RNAi-based therapeutics including delivery, stability and avoidance of activating immune responses. Though, the presence of evolutionary conserved miRs which were found to be present in all the species indicating their common origin and probably similarity in action infuse interest to researchers. Determining miRNA functions by applying gene knock out technique will be difficult. Many miRs are processed from introns of protein coding genes or multicistronic units containing multiple miRNA, thus making single miRNA knock-out animals difficult. Another big question among researchers are whether they operate through targeting single critical gene or do they act broadly by regulating minor effects on multiple targets, as suggested by evolutionary consideration. Parasite’s miRNAs also regulate host target genes and there is increasing evidence that pathogens exploit host miRNAs to aid replication and survival. miRNAs are essential for the rapid responses to environmental changes and the regulation of development involved in parasitism.

The main advantage of miRNA targeting based therapeutics over protein targeting is rather than intercepting a single target as in the case of selective protein inhibitors, miRNAs can regulate entire gene programs. Importantly, the outcome is tuning of target expression instead of blunting it, which should be less detrimental to healthy tissues. This demands for the identification of precise targets of each miRNA, which would considerably expand our understanding of parasitic helminths biology. Improved genome assembly/annotation together with comparative analysis will also help in target gene prediction through identification of conserved miRNA binding sites. However, to achieve this we must acknowledge that the relevant tools for miRNA target prediction are still lacking for all most every parasitic worm and helminths miRNA research needs major effort before we can use/target miRNA for treatment. It will be important to examine effects of helminth miRNAs on both parasite and host gene expression, any parasite-induced changes in host miRNA expression or copy number could also influence helminth tropism.

## Conclusion and Perspectives

Nowadays, miRNA is a hot issue in medical research field with huge potential. Despite the promising advancement in the applied areas of miRNA, several major theoretical and practical challenges remain to be resolved before this technique can be used in clinical application for treatment/diagnosis of infectious diseases. First, more basic investigations will be required to further examine the functional role of miRNAs in parasitic and other infectious diseases. Although, miRNAs can inhibit mRNAs by translational suppression or cleavage, others possible unknown mechanism could lead to translational or post-translation suppression. Second, the biological functions and packaging of extracellular miRNAs needs to be better understood. A number of studies had proposed that micro-vesicles containing miRNAs might be involved in the inflammatory site’s microenvironment. However, this will need more detailed knowledge about the detailed mechanism of miRNA specific function and action within complex disease pathways in one or more than one etiological agents, with tissue specific manner. Effort should be made to study the gene mutation frequency in miRNA and their target gene. Use of synthetic miRNA containing multiple binding sites or using small molecule inhibitor to reduce interaction between miRNAs and their targets will be useful to increase the efficiency and efficacy of miRNA based therapeutic tools. miRNA mimics, which are small, chemically modified double stranded RNA molecule that mimic endogenous mature miRNA can also be used to increase the target specificity and better delivery. Applying systems biology approaches, to describe role and activity of miRNA under conditions of homeostasis and disease may open new avenues. Another important avenues to be of immense importance is the delivery system, success of miRNA in therapeutic application relies on accurate delivery at the target site. A lot has to be done on this aspect. Finally before translation into clinical practice, all circulating miRNA findings require further steps of validation and an accurate standardization of all pre-analytical and analytical procedures, in order to control for all potential technical biases. Integration of data from whole genome atlas or miRBase will aid in future discoveries. Hence, use of miRNA for targeted therapy in near future is bright and impulsive at this moment; nevertheless, with ever increasing and rapid discoveries in this filed is certainly promising and have lots of potential. So, despite these caveats miRNA has strong potential to lead to therapeutic targeting of parasite cellular pathways as well as altering host-parasite interactions that influence parasite survival, which will be very beneficial for treatment of helminthic infections and personalized medicine for the pathology induced by helminthic infections. This calls for the complete understanding of receptor-targeted delivery of miRNA mimics or inhibitors to affected cells/tissues that may be critical for decreasing off-target effects, and for augmenting the utility of miRNAs.

## Author Contributions

NA, ST, AS, PM, AM, and AP designed the manuscript, NA, ST, AK, and AP wrote the manuscript. NA, PM, AM, and AP drafted and critically evaluated the manuscript.

## Conflict of Interest Statement

The authors declare that the research was conducted in the absence of any commercial or financial relationships that could be construed as a potential conflict of interest. The reviewers FFC, HLMG and handling Editor declared their shared affiliation, and the handling Editor states that the process nevertheless met the standards of a fair and objective review.

## Footnotes

1 www.mirbase.org/search.shtml
